# Supplementary material for: Hourglass Dirac chain metal in rhenium dioxide
Source: Nat Commun. 2017 Nov 29;8:1844. doi: 10.1038/s41467-017-01986-3 (PMC5705673; doi:10.1038/s41467-017-01986-3)
Supplement: Supplementary file 1 — Supplementary Information [file 41467_2017_1986_MOESM1_ESM.pdf]

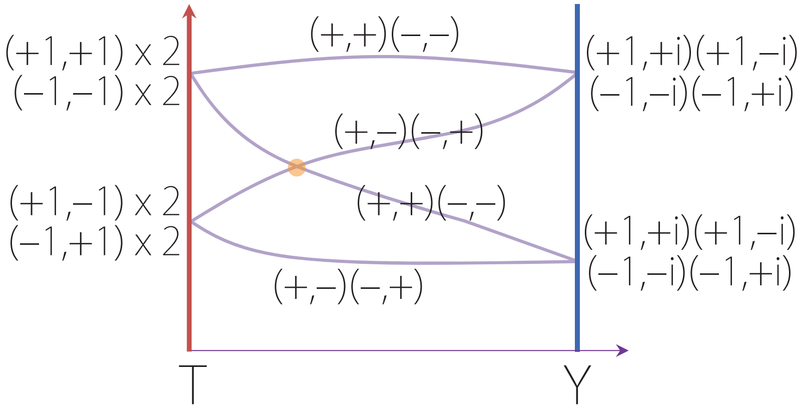

Supplementary Figure 1: **Hourglass Dirac point on T-Y.** Schematic figure showing the hourglass dispersion along T-Y. The states are four-fold degenerate at both T and Y. The numbers in each bracket indicate the eigenvalues (g_x, g_y) of the state. The neck crossing-point (orange dot) is the (isolated) four-fold-degenerate Dirac point on T-Y.

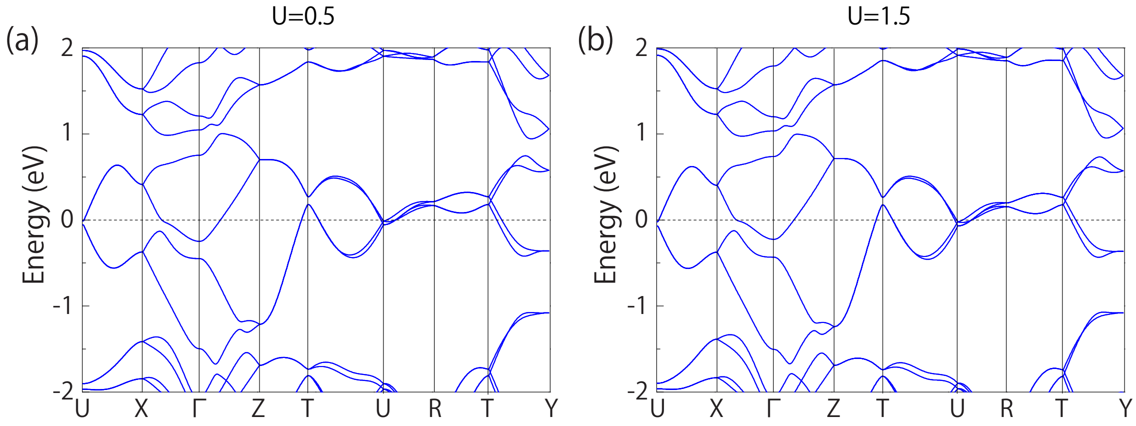

Supplementary Figure 2: **Effects of Hubbard U correction.** The GGA+U band structure of ReO_2 for (a) U=0.5 eV and (b) U=1.5 eV, showing the same qualitative features with GGA result.

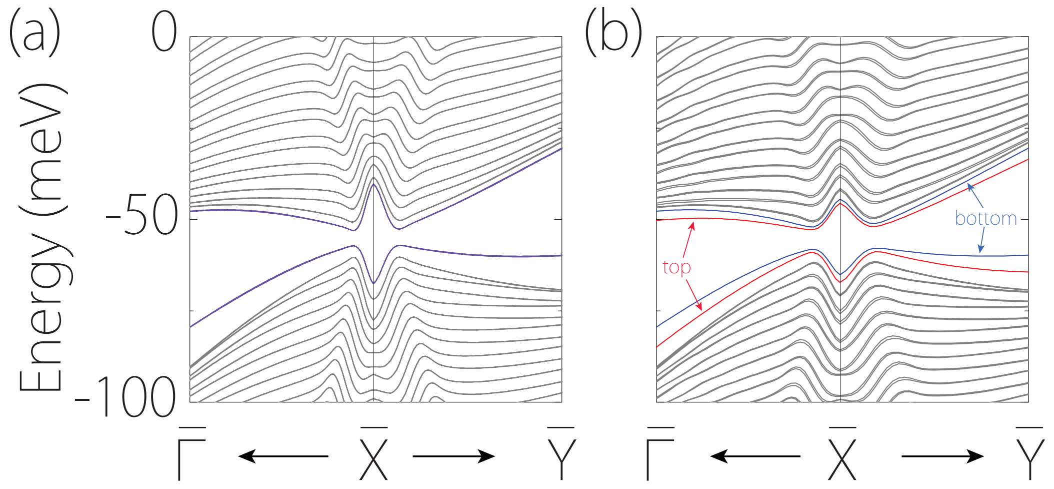

Supplementary Figure 3: **Spin-splitting in surface states.** The spin-splitting in surface states due to SOC can be explicitly seen in the result obtained from a slab calculation. (a) shows the result of a slab with (001) surface orientation and a thickness of 200 unit cells. From the spectrum and from the wave-function distribution, we verify that on each surface (top or bottom), there is one pair of spin-split surface bands (marked in purple color). In the obtained data, each purple line has a double-degeneracy because it includes states from both top and bottom surfaces. To demonstrate the point more explicitly, we apply a surface potential of -0.04 eV on the topmost layer, which is expected to shift the surface states on the top surface down in energy. This is confirmed by the calculation result plotted in (b). The top and bottom surface bands are now completely separated. One hence explicitly verifies that on each surface there is a pair of spin-split non-degenerate surface bands.

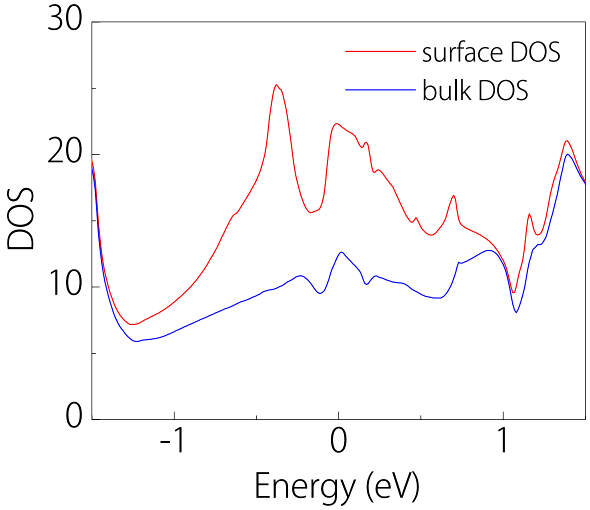

Supplementary Figure 4: **Comparison between bulk and surface density of states.** The plot shows the bulk density of states (blue curve) in comparison with the surface density of states (red curve) for the (001) surface. Their difference reflects the contribution from the drumhead-like surface states.

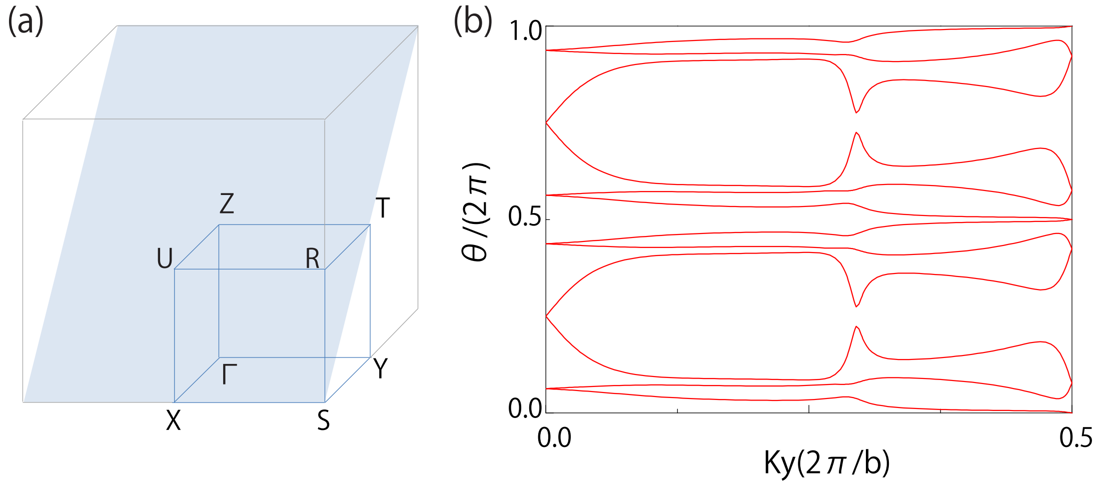

Supplementary Figure 5: $\mathbb{Z}_2$ **invariant for ZTS plane.** (a) A 2D $\mathbb{Z}_2$ invariant can be defined for the shaded plane containing points Z, T, and S of the bulk Brillouin zone. (b) The Wannier function centre evolution on ZTS plane, indicating a non-trivial $\mathbb{Z}_2$ invariant.

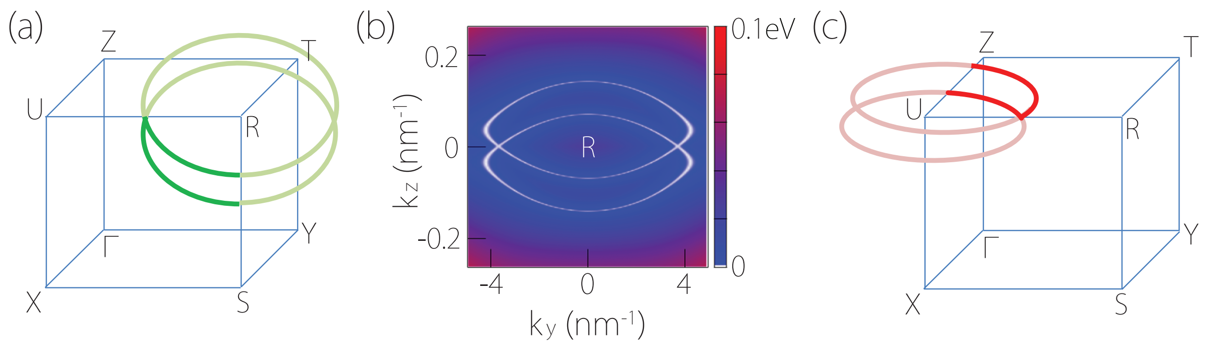

Supplementary Figure 6: **Transformation of Dirac chain under a Zeeman field.** (a,b) are results with a Zeeman field along the a-axis. The Dirac loop on the $k_z = \pi$ plane is removed, whereas the Dirac loop on the $k_x = \pi$ plane splits into two Weyl loops. (b) shows the shape of the two Weyl loops obtained from DFT calculation (with a Zeeman energy of 0.01 eV). (c) shows the result when the Zeeman field is along the c-axis. Then the original Dirac loop on the $k_z = \pi$ plane splits into two Weyl loops.

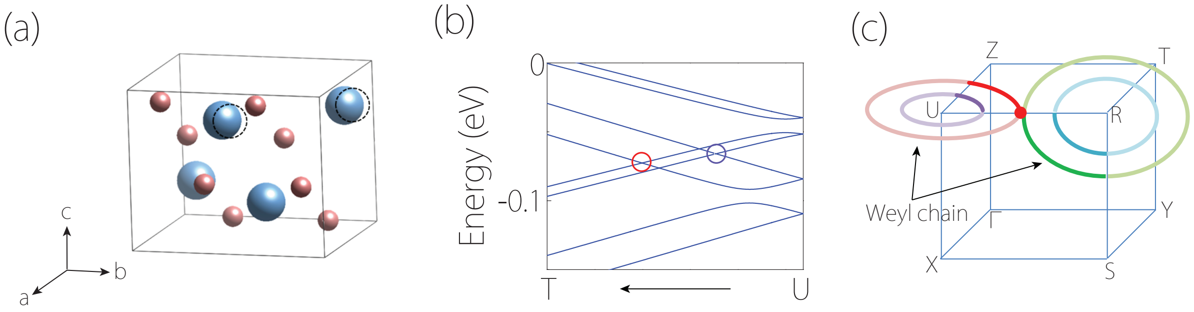

Supplementary Figure 7: **Transformation of Dirac chain under inversion symmetry breaking.** (a) We break the inversion symmetry by slightly displacing two Re atoms in the unit cell along the b-axis, such that $\mathcal{P}$ is broken while both $\widetilde{\mathcal{M}}_x$ and $\widetilde{\mathcal{M}}_z$ glide mirrors are preserved. The dashed circles indicate the original locations of the two atoms. (b) shows the calculated band structure along the path from U to T. One observes that the double-degeneracy of the bulk bands is lifted. However, the crossings between bands with opposite glide mirror eigenvalues are still protected, like the two crossings marked by the red and purple circles. Tracing these two crossings, we find that the original Dirac loop on the $k_z = \pi$ plane splits into two Weyl loops, as schematically shown in (c) (red and purple loops). Similar situation happens for the $k_x = \pi$ plane. As a result, the original Dirac chain transforms into a Weyl chain plus two isolated Weyl loops, as illustrated in (c).

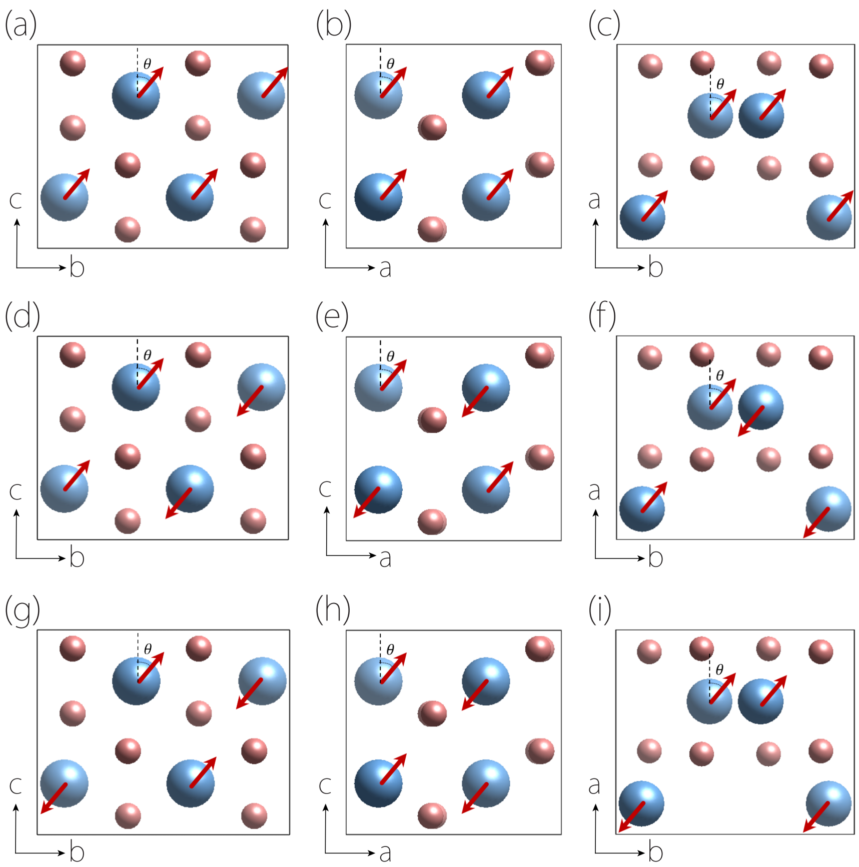

Supplementary Figure 8: **Test on magnetic ordered states.** We have performed test calculations of possible magnetic ordered stated for β-ReO_2. The figure shows the magnetic configurations that we studied. We have scanned the cases when the magnetic moments are in each of the three high-symmetry planes: (a,d,g) (100) plane; (b,e,h) (010) plane; and (c,f,i) (001) plane. (a-c) are for the ferromagnetic ordered states. (d-i) are for two types of antiferromagnetic ordered states.

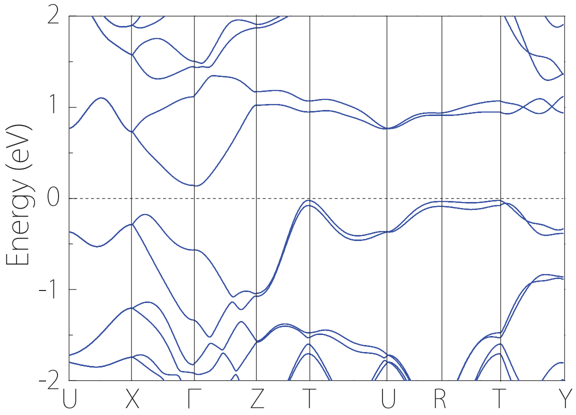

Supplementary Figure 9: **Band structure of the AFM state in Fig. 8(e).** The figure shows the band structure obtained from GGA+U+SOC approach for the AFM configuration in Fig. 8(e) with $\theta = 135°$ and with $U = 1.5$ eV.

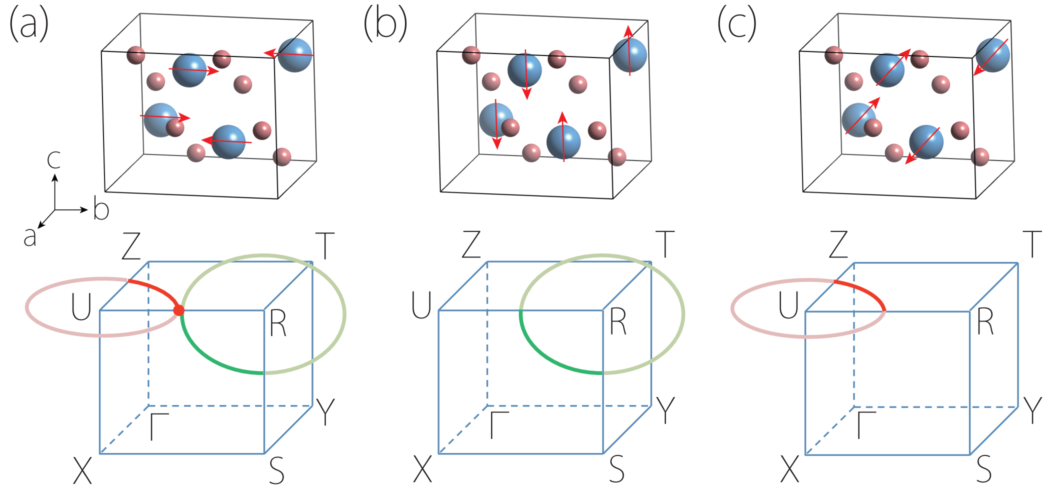

Supplementary Figure 10: **Preserved Dirac chain/loop under AFM configurations.** Schematic figure showing that the Dirac chain or loop could still be preserved under special antiferromagnetic (AFM) configurations where the magnetic moment is aligned with the high-symmetry direction. In each sub-figure, the upper panel shows the AFM configuration, and the lower panel illustrates the preserved band-crossings. (a) If the magnetic moment is aligned with the b-axis, the Dirac chain is preserved. (b,c) If the moment is aligned with the c or a axis, one Dirac loop is preserved.

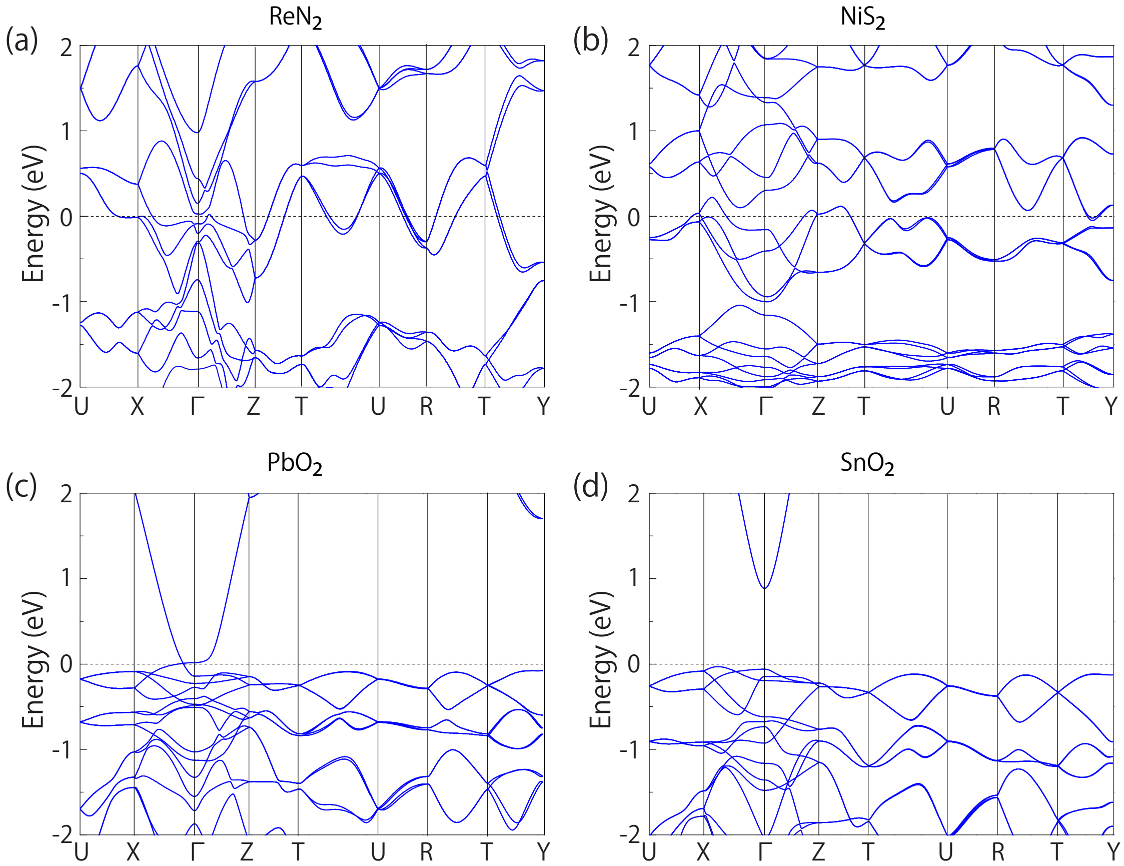

Supplementary Figure 11: **Band structures of other materials.** Band structure results of some other materials with space group No. 60: (a) ReN_2, (b) NiS_2, (c) PbO_2, and (d) SnO_2. Qualitatively the same features as ReO_2 can be observed, although the band splitting is quite small in some cases and the band-crossings are not close to the Fermi level.

Supplementary Note 1: Four-fold degeneracy along paths Z-T and T-R

In the main text, we have analyzed the mechanism for the four-fold degeneracy along the U-X line. Here we present the detailed analysis for the four-fold degeneracy occurring on the other two lines: Z-T and T-R.

First consider Z-T. It is invariant under the $\widetilde{\mathcal{M}}_z$ operation, so the states there can be chosen as eigenstates of $\widetilde{\mathcal{M}}_z$. Since

$$(\widetilde{\mathcal{M}}_z)^2 = T_{110}\overline{E}. \tag{1}$$

the eigenvalue of $\widetilde{\mathcal{M}}_z$ is $g_z = \pm i e^{-ik_y/2}$ for states on Z-T $(k_x = 0, k_z = \pi)$. From the commutation relation, From the relation,

$$\widetilde{\mathcal{M}}_z\mathcal{P} = T_{111}\mathcal{P}\widetilde{\mathcal{M}}_z = e^{-ik_x-ik_y-ik_z}\mathcal{P}\widetilde{\mathcal{M}}_z = -e^{-ik_y}\mathcal{P}\widetilde{\mathcal{M}}_z, \tag{2}$$

where in the last equality we specialize onto Z-T. Then

$$\widetilde{\mathcal{M}}_z(\mathcal{P}\mathcal{T}|g_z\rangle) = -e^{-ik_y}\mathcal{P}\mathcal{T}g_z|g_z\rangle = g_z(\mathcal{P}\mathcal{T}|g_z\rangle). \tag{3}$$

Thus the Kramers degenerate pair $|g_z\rangle$ and $\mathcal{P}\mathcal{T}|g_z\rangle$ share the same $\widetilde{\mathcal{M}}_z$ eigenvalue.

On the other hand, there is another anti-unitary symmetry $\widetilde{\mathcal{M}}_y\mathcal{T}$ on Z-T $[(\widetilde{\mathcal{M}}_y\mathcal{T})^2 = -1]$. Note that since

$$\widetilde{\mathcal{M}}_z\widetilde{\mathcal{M}}_y = -T_{01\bar{1}}\widetilde{\mathcal{M}}_y\widetilde{\mathcal{M}}_z = e^{-ik_y}\widetilde{\mathcal{M}}_y\widetilde{\mathcal{M}}_z, \tag{4}$$

on Z-T, we have

$$\widetilde{\mathcal{M}}_z(\widetilde{\mathcal{M}}_y\mathcal{T}|g_z\rangle) = e^{-ik_y}\widetilde{\mathcal{M}}_y\mathcal{T}g_z|g_z\rangle = -g_z(\widetilde{\mathcal{M}}_y\mathcal{T}|g_z\rangle). \tag{5}$$

The degenerate pair $|g_z\rangle$ and $\widetilde{\mathcal{M}}_y\mathcal{T}|g_z\rangle$ have opposite $\widetilde{\mathcal{M}}_z$ eigenvalues. Thus the four linearly independent states $\{|g_z\rangle, \mathcal{P}\mathcal{T}|g_z\rangle, \widetilde{\mathcal{M}}_y\mathcal{T}|g_z\rangle, \mathcal{P}\widetilde{\mathcal{M}}_y|g_z\rangle\}$ form a degenerate quartet, and the bands along Z-T are four-fold degenerate.

Similarly, for T-R line ($k_y = k_z = \pi$), we may choose the states to be $\widetilde{\mathcal{M}}_z$ eigenstates, with eigenvalues $g_z = \pm e^{-ik_x/2}$. From the relation

$$\widetilde{\mathcal{M}}_z\mathcal{P} = T_{111}\mathcal{P}\widetilde{\mathcal{M}}_z = e^{-ik_x-ik_y-ik_z}\mathcal{P}\widetilde{\mathcal{M}}_z = e^{-ik_x}\mathcal{P}\widetilde{\mathcal{M}}_z, \tag{6}$$

where in the last equality we specialize onto T-R, we find that

$$\widetilde{\mathcal{M}}_z(\mathcal{PT}|g_z\rangle) = e^{-ik_x}\mathcal{PT}g_z|g_z\rangle = g_z(\mathcal{PT}|g_z\rangle). \tag{7}$$

Thus the Kramers degenerate pair $|g_z\rangle$ and $\mathcal{PT}|g_z\rangle$ have the same $\widetilde{\mathcal{M}}_z$ eigenvalue. On the other hand, since T-R is also invariant under $\widetilde{\mathcal{M}}_y$ and $\{\widetilde{\mathcal{M}}_z, \widetilde{\mathcal{M}}_y\} = 0$ on T-R [from Eq.(4)], $\widetilde{\mathcal{M}}_y|g_z\rangle$ and $\mathcal{PT}\widetilde{\mathcal{M}}_y|g_z\rangle$ will have opposite $\widetilde{\mathcal{M}}_z$ eigenvalues. Thus the four states $\{|g_z\rangle, \mathcal{PT}|g_z\rangle, \widetilde{\mathcal{M}}_y|g_z\rangle, \mathcal{PT}\widetilde{\mathcal{M}}_y|g_z\rangle\}$ form the four-fold degeneracy.

Supplementary Note 2: Hourglass Dirac Point on T-Y path

Besides the Dirac chain (consisting of Dirac loops in $k_z = \pi$ and $k_x = \pi$ planes), there is also an hourglass fermion dispersion along T-Y. To see this, we note that T-Y ($k_x = 0$, $k_y = \pi$) is invariant under both $\widetilde{\mathcal{M}}_x$ and $\widetilde{\mathcal{M}}_y$. Since $[\widetilde{\mathcal{M}}_x, \widetilde{\mathcal{M}}_y] = 0$ on T-Y, the states there can be chosen as eigenstates of both operators:

$$(\widetilde{\mathcal{M}}_x, \widetilde{\mathcal{M}}_y)|g_x, g_y\rangle = (g_x, g_y)|g_x, g_y\rangle, \tag{8}$$

with the eigenvalues $g_x = \pm 1$, and $g_y = \pm i e^{-ik_z/2}$. By using the commutation relations between $\widetilde{\mathcal{M}}_i$ ($i = x, y$) and $\mathcal{P}$, one finds that

$$(\widetilde{\mathcal{M}}_x, \widetilde{\mathcal{M}}_y)\mathcal{PT}|g_x, g_y\rangle = (-g_x, -g_y)\mathcal{PT}|g_x, g_y\rangle. \tag{9}$$

This shows that the Kramers partner $\mathcal{PT}|g_x, g_y\rangle$ has opposite $\widetilde{\mathcal{M}}_i$ ($i = x, y$) eigenvalues compared with $|g_x, g_y\rangle$. For the end points T and Y, they are invariant under additional $\mathcal{T}$ symmetry. One notes that

$$(\widetilde{\mathcal{M}}_x, \widetilde{\mathcal{M}}_y)\mathcal{T}|g_x, g_y\rangle = (g_x, g_y)\mathcal{T}|g_x, g_y\rangle \tag{10}$$

at T, and

$$(\widetilde{\mathcal{M}}_x, \widetilde{\mathcal{M}}_y)\mathcal{T}|g_x, g_y\rangle = (g_x, -g_y)\mathcal{T}|g_x, g_y\rangle \tag{11}$$

at Y. Consequently, there must be a partner switching when going from T to Y on the path, as schematically shown in Fig. 1, forming the hourglass dispersion. One note that the degenerate doublet $(+-)(-+)$ and the doublet $(++)(--)$ have to cross in between. Because the two crossing branches have different eigenvalues, the crossing is stable and forms a Dirac point (there is a pair of such points due to time reversal symmetry). Note that for k-point deviate from T-Y, at least one of the $\widetilde{\mathcal{M}}_i$ $(i = x, y)$ symmetries is lost, hence the crossing point will generally be gapped. Thus there is no protected Dirac loop on the $k_y = \pi$ plane. This is indeed confirmed by our DFT result.

Supplementary Note 3: Band structure with Hubbard U correction

Since ReO_2 is $5d$ transition metal oxides, there could be electron correlation effects from Re-$5d$ orbitals. Here we use GGA+U method [1] (SOC included) to calculate the band structure of β-ReO_2 from $U = 0$ to $U = 1.5$ eV. We find that low-energy bands around Fermi level are not sensitive to the U correction (see Fig. 2 for results with $U = 0.5$ eV and $U = 1.5$ eV). The obtained results show qualitatively the same features, and the ground state is still a paramagnetic metal, which is consistent with the experimental finding [2,3].

Supplementary Note 4: Spin-splitting of surface states and surface density of states

The bulk bands of β-ReO_2 are spin-degenerate due to the presence of both $\mathcal{P}$ and $\mathcal{T}$ symmetries. However, at the surface, $\mathcal{P}$ is broken, so the surface bands will generally be spin-split under the strong SOC from Re-$5d$ orbitals. This spin-splitting cannot be easily observed from the surface projected spectrum as in Fig. 5(a) of the main text. Hence we perform a calculation with a slab geometry (SOC included). The result is shown in Fig. 3. We confirm that on a single surface (top or bottom) of the slab, there is one pair of spin-split non-degenerate drumhead surface bands.

The drumhead surface states make important contribution to the surface density of states. In Fig. 4, we plot the calculated surface density of states and compare it with the bulk density of states. One observes the additional contribution near the Fermi level due to the surface states. Here the surface state contribution does not form a very sharp peak, because of the non-negligible surface band dispersion (which is partly attributed to the large spin-splitting at the surface).

Supplementary Note 5: Bulk $\mathbb{Z}_2$ invariant for the ZTS plane

In the main text, we have shown the Fermi arcs on the (010) surface around point $\widetilde{\mathrm{T}}$, connecting the projections of two bulk Dirac points. We show that they are protected by a nontrivial $\mathbb{Z}_2$ invariant defined on a slant plane of Brillouin zone containing the points Z, T, and S. This plane does not intersect with any band crossings and is invariant under time reversal operation. The $\mathbb{Z}_2$ invariant is computed using the Wilson loop method [4–6]. The non-trivial invariant value, as shown in Fig. 5, requires the existence of a Kramers pair of surface states on the projection of this plane in the (010) surface Brillouin zone. Hence the surface Fermi arcs cannot be removed, and is protected by this nontrivial bulk $\mathbb{Z}_2$ invariant.

Supplementary Note 6: Transformation of Dirac chain under symmetry breaking

Here we show that under proper symmetry breaking, the hourglass Dirac chain metal can be transformed into other topological phases. We first consider the case with broken $\mathcal{T}$-symmetry via a Zeeman coupling. Physically, this may be achieved via magnetic doping or external magnetic field. As illustrated in Fig. 6, we find that when the Zeeman field direction is along the a (c) axis, then the original Dirac loop on the $k_x = \pi$ ($k_z = \pi$) plane will split into two Weyl loops, whereas the other Dirac loop will be gapped.

We also consider the case with broken $\mathcal{P}$-symmetry. Note that the $\mathcal{P}$-symmetry is in fact quite

robust against several types of strains, as we discussed in the main text. Here we break it by artificially displacing two Re atoms in the unit cell along the b-axis, as shown in Fig. 7(a). The breaking of $\mathcal{P}$ lifts the double degeneracy of each bulk band, hence the original Dirac band-crossings are removed. However, the deformation preserves the glide mirrors, such that the crossings between bands with opposite glide eigenvalues are still protected. As illustrated in Fig. 7(b,c), interestingly, the original Dirac chain gets transformed into a Weyl chain plus two isolated Weyl loops in this case.

Supplementary Note 7: Calculation of possible magnetic ordered state

The analysis in the main text is based on the paramagnetic phase. Experimentally, β-ReO_2 has been found to be a paramagnetic metal in a wide temperature range from room temperature down to liquid helium temperature (4.2 K) [2,3]. To be complete, we have also investigated the possible magnetic ordering of the system. With GGA+U method [1] (SOC included), we have tested a series of magnetic configurations including both ferromagnetic (FM) and antiferromagnetic (AFM) states, as shown in Fig. 8.

We find the following results. (i) The FM configurations are always energetically unfavorable (compared with nonmagnetic and AFM configurations). (ii) At $U = 0$ eV, all magnetic configurations (FM or AFM), will converge to the nonmagnetic result, with zero magnetic moments. (iii) AFM state appears to have a lower energy at large U. For $U = 1.5$ eV, we find that the AFM configuration in Fig. 8(e) with $\theta \approx 135°$ has the lowest energy, with an energy ~ 0.152 eV per unit cell lower than the nonmagnetic state and a magnetic moment $\sim 0.76\mu_B$ per Re site. The band structure result for this state shows an AFM insulator (see Fig. 9). Thus, from calculation, it is possible to have an AFM phase, but according to experiment, it can only appear at very low temperature at least below 4.2 K.

In addition, we find that if the AFM state indeed occurs in β-ReO_2, interestingly, the Dirac chain or loop band crossing may still be preserved when the magnetic moment is aligned along certain high-symmetry directions. This is schematically shown in Fig. 10 and confirmed by calculations. For example, the configuration in Fig. 10(a) preserves the combined $\mathcal{PT}$ symmetry, although the individual $\mathcal{P}$ and $\mathcal{T}$ are broken. Meanwhile, one can check that the glide mirrors $\widetilde{\mathcal{M}}_x$ and $\widetilde{\mathcal{M}}_z$ are also maintained, so that our argument in the main text on the symmetry-protection still applies and the Dirac chain is preserved. The cases in Fig. 10(b,c) can be analyzed in a similar way.

Supplementary Note 8: Other materials with the same space group

To further demonstrate that the hourglass Dirac chain is solely dictated by the space group symmetry, we calculate the band structures of other materials with the same space group (No. 60). The results of a few examples are shown in Fig. 11, including ReN_2, NiS_2, PbO_2 and SnO_2. One indeed observes that same features as discussed in the main text also appear in these results, although the band splitting is quite small in some cases (e.g. in SnO_2. However, compared with ReO_2, the interesting band-crossings in these materials are not close to the Fermi level, making them not as good as ReO_2 for exhibiting the Dirac chain physics.

Supplementary References

1. Dudarev, S. L., Botton, G. A., Savrasov, S. Y., Humphreys, C. J. & Sutton, A. P. Electron-energy-loss spectra and the structural stability of Nickel oxide: An LSDA+U study. *Phys. Rev. B* **57**, 1505–1509 (1998).

2. Goodenough, J. B., Gibart, P. & Brenet, J. Magnetic and electric properties of $\mathrm{ReO_2}$ – theoretical interpretation. *CR Hebd. S 'eances Acad. Sci.* **261**, 2331–2343 (1965).

3. Rogers, D. B., Shannon, R. D., Sleight, A. W. & Gillson, J. L. Crystal chemistry of metal dioxides with rutile-realted structures. *Inorg. Chem.* **8**, 841–849 (1969).

4. Yu, R., Qi, X. L., Bernevig, A., Fang, Z. & Dai, X. Equivalent expression of $\mathbb{Z}_2$ topological invariant for band insulators using the non-abelian Berry connection. *Phys. Rev. B* **84**, 075119 (2011).

5. Soluyanov, A. A. & Vanderbilt, D. Wannier representation of $\mathbb{Z}_2$ topological insulators. *Phys. Rev. B* **83**, 035108 (2011).

6. Q. S. Wu, S. N. Zhang, H.-F. Song, M. Troyer & A. A. Soluyanov. Wannier-Tools: An open-source software package for novel topological materials. Preprint at http://arxiv.org/abs/1703.07789 (2017)
